# A modified frailty index predicts complication, readmission, and 30-day mortality following the revision total hip arthroplasty

**DOI:** 10.1186/s42836-024-00232-8

**Published:** 2024-02-04

**Authors:** David Momtaz, Shawn Okpara, Armando Martinez, Tucker Cushing, Abdullah Ghali, Rishi Gonuguntla, Travis Kotzur, Anthony Duruewuru, Madison Harris, Ali Seifi, Melvyn Harrington

**Affiliations:** Department of Orthopaedics, Baylor College of Medicine, Houston, TX 77030 USA

**Keywords:** Revision, Total hip arthroplasty, Risk assessment, Modified frailty index, MFI, Operative risk factors, Surgery, Orthopaedics, Orthopedics

## Abstract

**Introduction:**

This study aimed to develop a modified frailty index (MFI) to predict the risks of revision total hip arthroplasty (THA).

**Methods:**

Data from the American College of Surgeons - National Surgical Quality Improvement Program were analyzed for patients who underwent revision THA from 2015 to 2020. An MFI was composed of the risk factors, including severe obesity (body mass index > 35), osteoporosis, non-independent function status prior to surgery, congestive heart failure within 30 days of surgery, hypoalbuminemia (serum albumin < 3.5), hypertension requiring medication, type 1 or type 2 diabetes, and a history of chronic obstructive pulmonary disease or pneumonia. The patients were assigned based on the MFI scores (MFI0, no risk factor; MFI1, 1–2 risk factors; MFI2, 3–4 risk factors; and MFI3, 5+ risk factors). Confidence intervals were set at 95% with a *P* value less than or equal to 0.05 considered statistically significant.

**Results:**

A total of 17,868 patients (45% male, 55% female) were included and had an average age of 68.5 ± 11.5 years. Odds of any complication, when compared to MFI0, were 1.4 (95% CI [1.3, 1.6]) times greater for MFI1, 3.2 (95% CI [2.8, 3.6]) times greater for MFI2, and 10.8 (95% CI [5.8, 20.0]) times greater for MFI3 (*P* < 0.001). Odds of readmission, when compared to MFI0, were 1.4 (95% CI [1.3, 1.7]) times greater for MFI1, 2.5 (95% CI [2.1, 3.0]) times greater for MFI2, and 4.1 (95% CI [2.2, 7.8]) times greater for MFI3 (*P* < 0.001).

**Conclusion:**

Increasing MFI scores correlate with increased odds of complication and readmission in patients who have undergone revision THA. This MFI may be used to predict the risks after revision THA.

## Background

Primary total hip arthroplasty (THA) remains incredibly cost-effective, and the patient satisfaction rate is 90% [1, 2]. The demand for a THA will increase as the general population gets older, and despite improvements in surgical technique, materials, and physical therapy, half of these prosthetics will need replacement after 25 years [3], secondary to instability and dislocation, hardware loosening, and infection [4, 5].

It is projected that the amount of THAs performed will increase by 71% (635,000 procedures) by 2030, and up to 1.23 million procedures by 2060, with revisions increasing at a similar rate [6]. Further compounding the demand for these surgeries, national trends in comorbidities may be influencing how early patients need THA [7]. High rates of obesity, are expected to add to this growing demand for THAs at a younger age [8–10]. Between 2000 and 2014, the mean age for THA decreased by almost 2 years (66.3 to 64.9). The earlier a patient gets a primary THA, the more likely they are to require a revision [6, 11].

Some studies estimate that between 60%–88% of people aged above 65 years have at least one comorbidity [12]. Centers for Disease Control and Prevention data show similar statistics with estimates of 86% of United States adults over 60 years having at least one chronic condition. Comorbidities such as congestive heart failure, diabetes, chronic pulmonary disease, and hypertension often lead to an increased risk of perioperative complications [13]. Beyond the issue of age are also specific comorbidities prudent in the consideration of more challenging surgeries like revision arthroplasties [14, 15].

It is imperative to assess perioperative risks. To do so, surgeons may utilize a comorbidity-based risk stratification tool, or modified frailty index (MFI). Previous studies have shown that these tools are useful predictors for morbidity, mortality, readmission, adverse discharge, postoperative infections, and a plethora of other complications [16, 17]. While other studies have looked at MFIs for THA, none have been studied in revision THA cases [18–20]. The impact of specific comorbidities important for revisions remains to be discovered [21]. Thus, the addition of factors such as hypoalbuminemia and osteoporosis to the 5-item MFI may be prudent [14, 15, 22].

We designed an 8-item MFI to stratify preoperative risks for patients undergoing revision THA. This study aimed to develop a modified frailty index (MFI) to predict the risks for revision total hip arthroplasty (THA). We hypothesize that the MFI can be used to predict the risks of complications after revision THA.

## Methods

We conducted a retrospective analysis of the American College of Surgeons-National Surgical Quality Improvement Program database on patients undergoing revision THA between 2016 and 2020. A modified frailty index (MFI) was created from 8 variables: non-independent functional status prior to surgery, severe obesity (body mass index > 35), type I or type II diabetes, congestive heart failure within 30 days of surgery, hypoalbuminemia (albumin < 3.5 mg/dL), hypertension requiring medication, history of chronic obstructive pulmonary disease or pneumonia, and osteoporosis. Independent functional status was defined as being able to perform activities of daily living alone. The presence of each variable was scored as 1, and the MFI was calculated by summing the total point of each patient (range: 0–8). Higher MFI scores indicated increased frailty.

Patient variables collected included demographic information, American Society of Anesthesiologists score, smoking history, and medical comorbidities including congestive heart failure, hypertension, chronic obstructive pulmonary disorder, diabetes, and dialysis-dependent kidney disease. Body mass index (BMI) was calculated from patient height and weight. We included all patients who were older than 50 and underwent revision total hip arthroplasty with matching Current Procedural Terminology codes 27134, 27137 and 27138. Patients were excluded if the data collection was missing or had obvious errors. Following the exclusion of incomplete and missing data, patients were then sorted into 4 groups based on their MFI scores: MFI0 (score of 0), MFI1 (score of 1–2), MFI 2 (score of 3–4), MFI3 (score > 4).

### Statistical analysis

The Statistical Package for the Social Sciences Version 13.0 (SPSS, Inc., Chicago, IL, USA) was utilized to analyze the data, with the G*Power Statistics tool used to perform power analysis. Confidence intervals were set at 95% with a *P* value less than or equal to 0.05 considered statistically significant.

Between-group analysis was performed to compare complication rates and means of various variables. Of note, readmissions and reoperations were defined to be within 30 days after operation. Multiple linear and logistic regression models were created to determine the relationship between the MFI category and postoperative complications and resource utilization outcomes while controlling for sex, race, ethnicity, age, and BMI. Continuous data were reported as means ± standard deviations); standard errors were given where appropriate. Categorical results were presented as counts with column percentages. All data were initially analyzed to ensure that appropriate statistical assessments were used and that variables satisfied the assumptions and requirements of each statistical test. Normally distributed data were analyzed using independent sample *t*-tests. The Wilcoxon rank-sum test was employed for non-normally distributed data. Fisher’s Exact Test or the Chi-Square test with Kendall Tau was utilized to compare categorical variables. Both multiple linear and logistic regression models were analyzed to ensure all assumptions were met. Where appropriate, residuals were assessed for normal distribution and no multicollinearity was observed. All variables in the multiple linear logistic regression model were first run separately to ensure no artifact *P* values were present and that all effect sizes were reported honestly.

## Results

We identified 17,868 patients who underwent revision THA between 2015–2020. The mean age of patients was 66.68 years. 55% of the patients were female, and 8% of patients were black. A detailed demographic breakdown of each MFI category can be found in Table 1. The breakdown of patients in each MFI group was as follows: 4,849 patients were in MFI0, 11,071 patients were in MFI1, 1,883 patients were in MFI2, and 65 patients were in MFI3 group.

**Table 1:** Demographics and characteristics of study population

	**Categories of MFI**							
	**MFI 0 (0 RF)**		**MFI 1 (1–2 RF)**		**MFI 2 (3–4 RF)**		**MFI 3 (5+ RF)**	
	**Mean**	**SD**	**Mean**	**SD**	**Mean**	**SD**	**Mean**	**SD**
Age	63	13	68	12	69	11	74	10
BMI	26.9	4.16	30.3	6.84	34.8	8.25	38.2	9.8
Sex	Count	Row%	Count	Row%	Count	Row%	Count	Row%
Male	2,131	26.2%	5,033	62.0%	928	11.4%	29	0.4%
Female	2,718	27.9%	6,038	61.9%	955	9.8%	36	0.4%
Race	Count	Row%	Count	Row%	Count	Row%	Count	Row%
Not Black	4,561	27.8%	10,111	61.7%	1,654	10.1%	60	0.4%
Black	288	19.4%	960	64.8%	229	15.5%	5	0.3%
Status	Count	Row%	Count	Row%	Count	Row%	Count	Row%
Elective	4,178	29.8%	8,622	61.4%	1,208	8.6%	32	0.2%
Non-Elective	671	17.5%	2,449	64.0%	675	17.6%	33	0.9%

*n* = 17,868, *MFI* Modified Frailty Index, *RF* risk factors, *SD* standard deviation, Row%: percent of row demographic per listed category

**Fig. 1:**
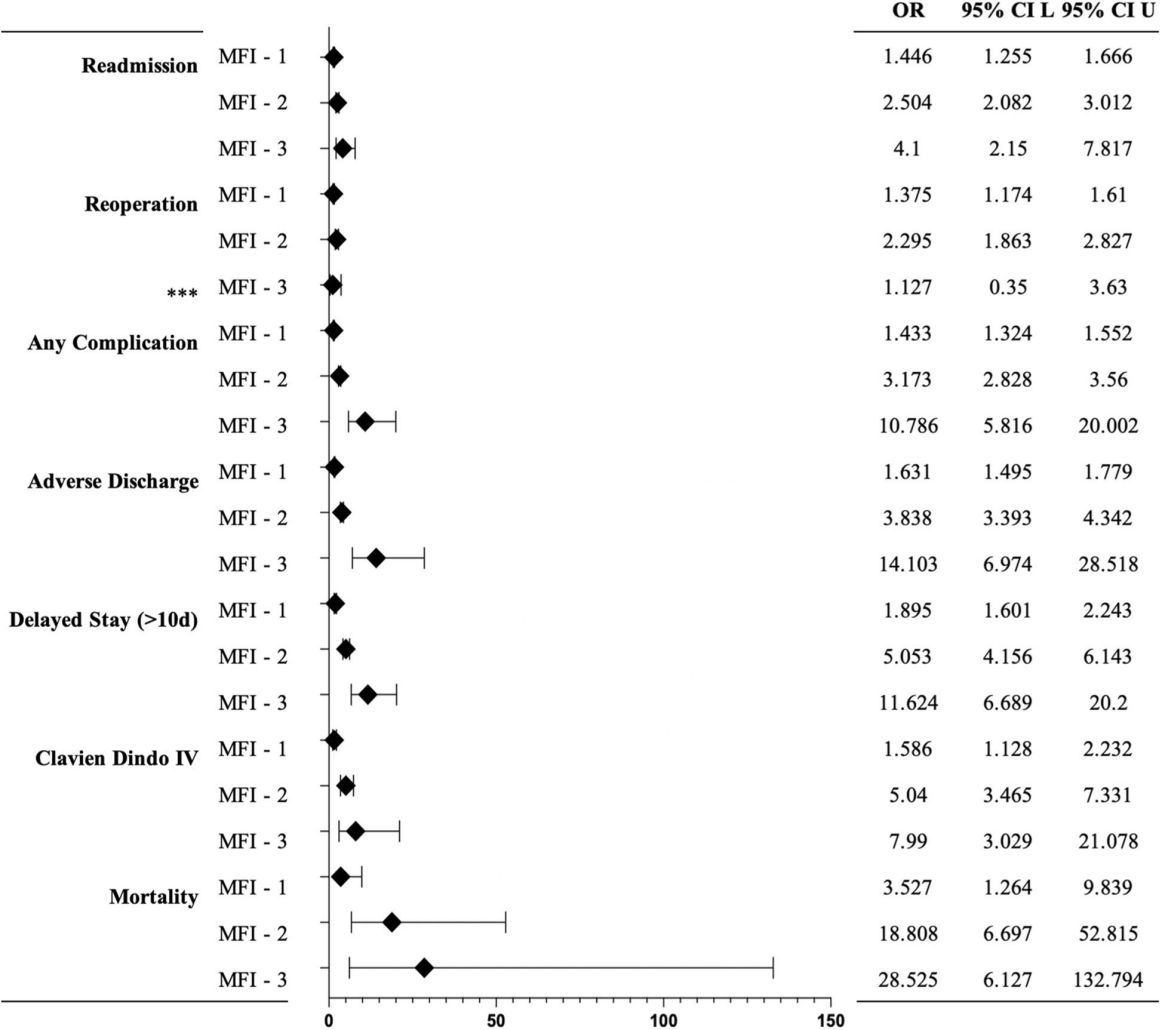
Odd ratios of general complications per MFI group. *n* = 17,868. MFI: Modified Frailty Index, OR: odds ratio, CI: confidence interval, L: Lower bound of the confidence interval, U: Upper bound of the confidence interval. Odds ratio for each MFI group for each complication. All complications presented are significant at *P* < 0.001. 95% CI (***unless otherwise indicated)

When compared to the MFI0 group, the odds of readmission were 1.446 times higher in the MFI1 group (*P* < 0.001), 2.504 times higher in the MFI2 group (*P* < 0.001), and 4.100 times higher in the MFI3 group (*P* < 0.001). Additionally, the odds of reoperation were 1.375 times higher in the MFI1 group (*P* < 0.001), and 2.295 times higher in the MFI2 group (*P* < 0.001) when compared to the MFI0 group. However, they were not significantly higher in the MFI3 group (*P* < 0.842) when compared to the MFI0 group. The odds of having any complication were 1.433 times higher in the MFI1 group (*P* < 0.001), 3.173 times higher in the MFI2 group (*P* < 0.001), and 10.786 times higher in the MFI3 group (*P* < 0.001) when compared to the MFI0 group. This information can be found in Tables 2, 3 and Fig. 1.

**Table 2:** Odd ratios of general complications per MFI group

		**OR**	**95% CI L**	**95% CI U**
Readmission	MFI-1	1.4	1.3	1.7
	MFI-2	2.5	2.1	3.0
	MFI-3	4.1	2.2	7.8
Reoperation ^***^	MFI-1	1.4	1.2	1.6
	MFI-2	2.3	1.9	2.8
	MFI-3	1.1	0.4	3.6
Complication	MFI-1	1.4	1.3	1.6
	MFI-2	3.2	2.8	3.6
	MFI-3	10.8	5.8	20.0
Adverse discharge	MFI-1	1.6	1.5	1.8
	MFI-2	3.8	3.4	4.3
	MFI-3	14.1	7.0	28.5
Delayed stay (>10 day)	MFI-1	1.9	1.6	2.2
	MFI-2	5.1	4.2	6.1
	MFI-3	11.6	6.7	20.2
Clavien dindo IV	MFI-1	1.6	1.1	2.2
	MFI-2	5.0	3.6	7.3
	MFI-3	8.0	3.0	21.1
Mortality	MFI-1	3.5	1.3	9.8
	MFI-2	18.8	6.7	52.8
	MFI-3	28.5	6.1	132.8

*n* = 17,868, *MFI* Modified Frailty Index, *OR* odds ratio, *CI* confidence interval, *L* Lower bound of the confidence interval, *U* Upper bound of the confidence interval. Odds ratio for each MFI group for each complication. All complications presented are significant at *P* < 0.001. 95% CI (***unless otherwise indicated)

**Table 3:** Odd ratios of system-based complications per MFI group

		**OR**	**95% CI L**	**95% CI U**
Renal	MFI-1	4.5	1.8	11.2
	MFI-2	13.8	5.3	36.2
	MFI-3	49.0	11.2	215.3
Wound	MFI-1	1.6	1.4	1.7
	MFI-2	3.3	2.8	3.8
	MFI-3	8.1	4.8	13.7
Hematological	MFI-1	1.3	1.2	1.5
	MFI-2	2.4	2.1	2.8
	MFI-3	5.2	3.1	8.7
Pulmonary	MFI-1	5.6	2.6	12.2
	MFI-2	32.2	14.9	70.0
	MFI-3	119.3	44.0	323.8

*n* = 17,868. *MFI* Modified Frailty Index, *OR* odds ratio, *CI* confidence interval, *L* Lower bound of the confidence interval, *U* Upper bound of the confidence interval. Odds ratio for each MFI group for each complication. All complications presented are significant at *P* < 0.001

**Fig. 2:**
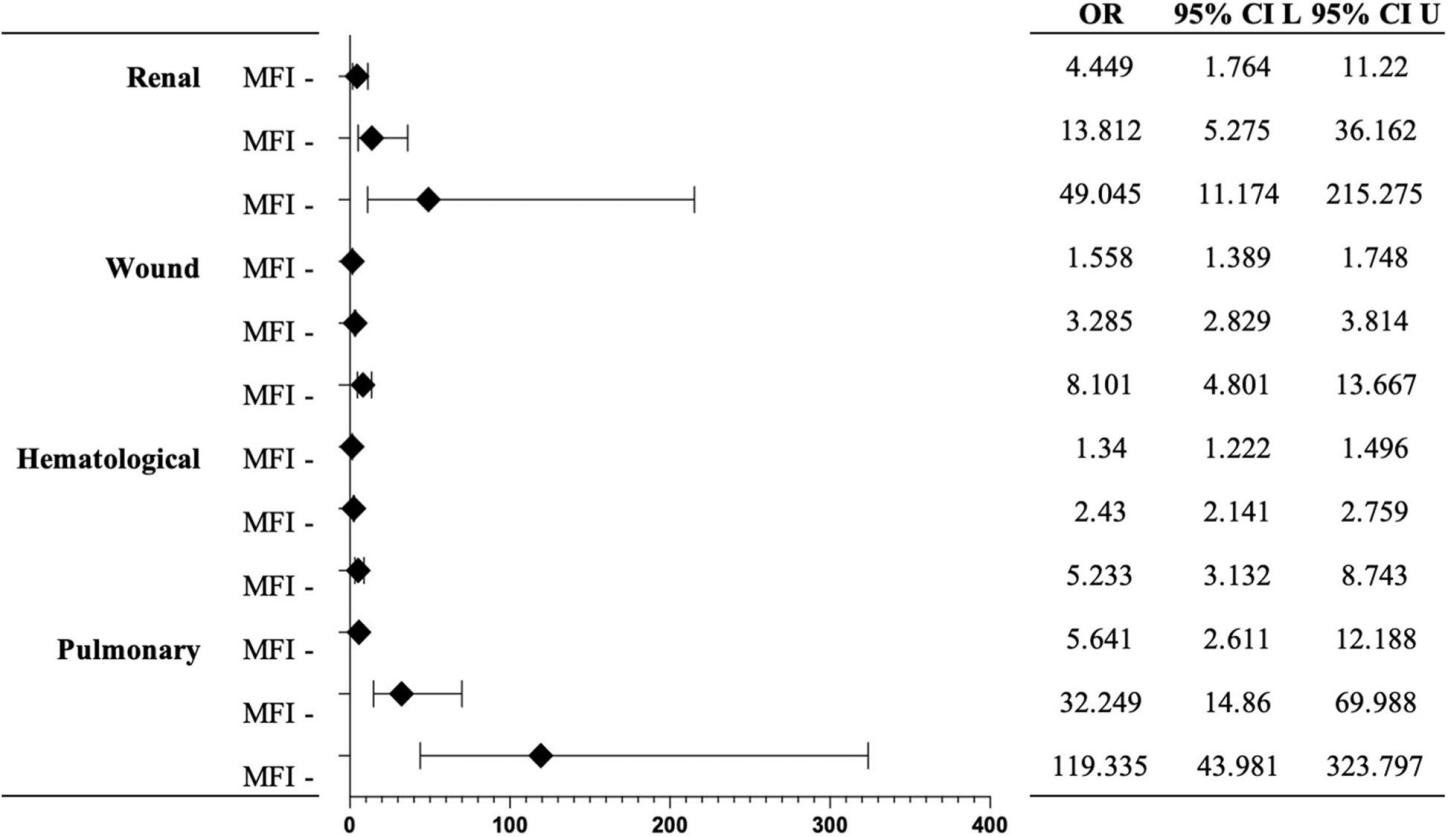
Odd ratios of system-based complications per MFI group. *n* = 17,868. MFI: Modified Frailty Index, OR: odds ratio, CI: confidence interval, L: Lower bound of the confidence interval, U: Upper bound of the confidence interval. Odds ratio for each MFI group for each complication. All complications presented are significant at *P* < 0.001. 95% CI (***unless otherwise indicated)

The odds of having an adverse discharge were 1.631 times higher in the MFI1 group (*P* < 0.001), 3.838 times higher in the MFI2 group (*P* < 0.001), and 14.103 times higher in the MFI3 group (*P* < 0.001) when compared to the MFI0 group, and the odds of delayed stay longer than 10 days was 1.895 times higher in the MFI1 group (*P* < 0.001), 5.053 times higher in the MFI2 group (*P* < 0.001), and 11.624 times higher in the MFI3 group (*P* < 0.001). Finally, when compared to the MFI0 group, the odds of mortality were 3.527 times higher in the MFI1 group (*P* < 0.001), 18.808 times higher in the MFI2 group (*P* < 0.001), and 28.525 times higher in the MFI3 group (*P* < 0.001). This information can be found in Fig. 2.

## Discussion

Our 8-item MFI can be used to predict postoperative outcomes in patients undergoing revision THA when controlling for age and race. Uniquely, including osteoporosis and hypoalbuminemia sets our MFI apart and can be used to assess frailty preoperatively.

Regarding BMI and age, studies have found conflicting data on using these indices to predict postoperative complications. Lubbeke et al. found that increased BMI was related to increased rates of adverse events following revision THA in their study of 204 patients from their hospital [23]. However, Roth et al. conducted a retrospective analysis of 18,866 patients from the American College of Surgeons-National Surgical Quality Improvement Program database and found that BMI was not significantly associated with increased rates of reoperation or readmission following revision THA when controlling for other variables [24]. Koenig et al. found that increased age was related to higher rates of major adverse events (such as arrhythmia, pulmonary embolism, mortality, etc.) as well as higher rates of minor adverse events (such as superficial infection, deep vein thrombosis, ileus) in their retrospective analysis of 306 patients with 322 revision THAs at their institution [25]. Badarudeen et al. studied Medicare patients undergoing revision THA between 1998–2011 and found that increased age was related to increased rates of thromboembolic events and mortality [26]. Although there is evidence to support that BMI and age function well as simple preoperative heuristics for orthopaedic surgeons to have a rough idea of the risks these conditions carry in patients undergoing revision THA, neither of these are predictive of patient complication rates postoperatively.

Instead of BMI and age alone, frailty has been adopted as a multi-factorial index to predict postoperative complication rates. Johnson et al. constructed a frailty index utilizing 32 items recorded in their electronic medical registry, including BMI, 17 chronic conditions, and the ability to perform 14 activities of daily living, and found that a higher preoperative frailty index was associated with increased mortality and perioperative complications [27]. A unique strength of this index is that it includes many activities of daily living in determining frailty; however, their index was only validated against cases from a single institution which reduces the generalizability of their findings. Several studies have found that 5 and 11-item MFIs are effective predictors of postoperative complications in patients undergoing primary THA [18–20]. When specifically looking at revision arthroplasty, the literature is less robust. However, one study explored an age-adjusted modified frailty index. This added an age component to the 5-Item modified frailty index and ultimately has shown promise in predicting complications [21]. Insightful on the effect of age, our MFI approaches this issue from the perspective of more specific comorbidities to revision outcomes outside of age.

Besides frailty, few indices have been developed to predict patient risk for adverse events following revision THA. Meyer et al. utilized the Hospital Frailty Risk Score, a validated score predicting outcomes in geriatric patients, and found that this score is effective when predicting outcomes following revision THA [28]. However, the score is only validated for geriatric patients. As younger populations have started to utilize THA more frequently, additional tools to stratify risk should be utilized that are effective in younger patients to inform clinical decision-making [22, 29].

Our 8-item MFI carries some unique advantages due to the inclusion of hypoalbuminemia and osteoporosis. The first is the inclusion of hypoalbuminemia as a marker of malnutrition. Wilson et al. demonstrated that frail patients with hypoalbuminemia had increased rates of adverse postoperative outcomes following elective THA, and including hypoalbuminemia makes our 8-item MFI more predictive than the 5-item MFI [14]. Osteoporosis can contribute to hip fractures and is another critical aspect of a frailty index used for outcomes following orthopaedic surgery [15]. There are numerous advantages to utilizing our 8-item MFI compared to other indices.

Out study has limitations. First, we included non-elective cases which in a revision THA may be a result of infection. Additional research is needed to investigate the usefulness of our MFI to predict postoperative outcomes in elective vs. non-elective groups for revision THA. We are also interested in comparing our MFI to other indices such as the 32-item, 5-item, and 11-item indices using the same patient population to help further guide clinical judgement for revision THA.

The retrospective nature of this study predisposes it to several disadvantages. However, this also enables us to utilize the American College of Surgeons-National Surgical Quality Improvement Program database which includes a large, representative sample of patients from around the United States. This allows us to identify trends that may not be significant in smaller samples. Additionally, this database does not include information about the detailed clinical course that each patient had. In a revision THA, where patients must have undergone prior THA, this information would be useful in determining the need for revision surgery. The lack of detailed clinical information denies the opportunity to investigate the impact that surgical approach, rehabilitation management, length of follow-up, and other patient variables may have on played in the results that we identified.

## Conclusion

Our 8-item MFI is highly predictive of postoperative complications, readmission, reoperation, delayed hospital stays, and mortality. This new 8-item MFI addresses a need for a predictive and clinically useful frailty index for outcomes following a revision THA.

## Data Availability

The data set used and analyzed during the current study are available in the National Surgical Quality Improvement Program (NSQIP) database between 2015 and 2021.
